# Formal representation of complex SNOMED CT expressions

**DOI:** 10.1186/1472-6947-8-S1-S9

**Published:** 2008-10-27

**Authors:** Stefan Schulz, Kornél Markó, Boontawee Suntisrivaraporn

**Affiliations:** 1 Institute of Medical Biometry and Medical Informatics, University Medical Center, Freiburg, Germany; 2 AVERBIS GmbH, Freiburg, Germany; 3 Master Program in Health Technology, Pontificial Catholic University of Paraná, Curitiba, Brazil; 4 Department of Computer Science, Technical University Dresden, Germany

## Abstract

**Background:**

Definitory expressions about clinical procedures, findings and diseases constitute a major benefit of a formally founded clinical reference terminology which is ontologically sound and suited for formal reasoning. SNOMED CT claims to support formal reasoning by description-logic based concept definitions.

**Methods:**

On the basis of formal ontology criteria we analyze complex SNOMED CT concepts, such as "Concussion of Brain with(out) Loss of Consciousness", using alternatively full first order logics and the description logic ℰℒ.

**Results:**

Typical complex SNOMED CT concepts, including negations or not, can be expressed in full first-order logics. Negations cannot be properly expressed in the description logic ℰℒ underlying SNOMED CT. All concepts concepts the meaning of which implies a temporal scope may be subject to diverging interpretations, which are often unclear in SNOMED CT as their contextual determinants are not made explicit.

**Conclusion:**

The description of complex medical occurrents is ambiguous, as the same situations can be described as (i) a complex occurrent *C *that has *A *and *B *as temporal parts, (ii) a simple occurrent *A' *defined as a kind of A followed by some *B*, or (iii) a simple occurrent *B' *defined as a kind of *B *preceded by some *A*. As negative statements in SNOMED CT cannot be exactly represented without a (computationally costly) extension of the set of logical constructors, a solution can be the reification of negative statments (e.g., "Period with no Loss of Consciousness"), or the use of the SNOMED CT context model. However, the interpretation of SNOMED CT context model concepts as description logics axioms is not recommended, because this may entail unintended models.

##  Introduction

Improving semantic interoperability by a structured representation of clinical procedures, findings and diseases constitutes the main rationale for the development of clinical terminologies and classification systems. The applicability of a clinical reference terminology such as SNOMED CT is critically coupled to the extent of how it can express the language-independent meaning of complex and often idiosyncratic medical terms in a principled way. Such pre-coordinated terms, which are typical for medical classification systems, should be definable in terms of the primitives provided by the reference terminology.

SNOMED CT (Clinical Terms) is a clinical terminology which was built by merging, restructuring, and enhancing the previous SNOMED version RT (Reference Terminology) with the former UK Read Codes. Already SNOMED RT had claimed to be a "set of concepts and relationships that provides a common reference point for comparison and aggregation of data about the entire health care process" [1,2]. To fulfill this requirement, SNOMED CT contains now (May 2008) over half a million concepts. SNOMED CT relational statements are provided as concept – relation – concept triples. However, there is no clear explanation about what SNOMED CT "concepts" and "relations" exactly stand for. SNOMED CT "concepts", for instance, encompass individuals like *Denmark*, *Greater London*, *Association of Anesthesia Clinical Directors*, etc., and relations are allowed to link both concepts and individuals. We therefore prefer the term "class" wherever categories of individual entities are meant (which constitutes the standard case). Furthermore, we distinguish *specialization *relations which hold between classes (here the subclass-superclass relation *is-a*) from *instantiation *relations which relate individuals to their categorizing classes, using the instantiation relation **inst**. Moreover, domain-specific relations, such as "associated morphology" or "is part of", are intuitively used to relate individuals. These are to be differentiated from the former two relations and are usually referred to as *roles*. Wherever a role (domain-specific relation) is used in a class definition, it needs to be quantified, as shown below.

### Logical stipulations

SNOMED RT and SNOMED CT (partially) follow a formal semantics based on the description logic specification KRSS [3]. The standard semantics of the Krss specification has been enhanced in SNOMED CT to cater for so-called right-identity rules which are shown essentially important for certain part-whole reasoning tasks.

Description logics are a family of decidable fragments of the first-order logic, which have a clean and intuitive syntax (without the need for free variables), cf. [4]. They have become increasingly popular, in particular, by the W3C recommendation of the Semantic Web language OWL-DL [5], several description logic based ontology editors, such as Protégé [6], and description logic reasoners such as CEL and FaCT++ which already proved feasibly useful in classifying SNOMED CT [7]. The description logic ℰℒ – the underlying logical formalism for the SNOMED CT terminology – provides *conjunctions *and *existential quantifications *which can be conceived as building blocks for complex class descriptions. Conjunctions are most easily to be understood as intersections of sets. For instance, the expression FractureOfBone ⊓ LegInjury denotes the intersection of all entities belonging to the class FractureOfBone with those belonging to the class LegInjury. The resulting class LegFracture therefore contains all injuries which are both leg injuries and bone fractures. To give an example for existential quantification, ∃part-of.Body denotes the class of all entities which are part of some body. The equivalence between a named class *A *and a class description *D *can be asserted into the terminology by using a class definition (denoted by ≡). Such a class definition provides both *necessary *and *sufficient *conditions, specified in terms of *D*, for being qualified as instance of the class *A*, e.g. BodyPart ≡ ∃part-of.Body. When only necessary conditions are known for a certain named class, a primitive class definition (denoted by the subsumption operator ⊑) can be used instead. The SNOMED CT class definitions are partly primitive, partly fully defined, with a few additional axioms on roles, e.g. part-of is declared transitive.

### Ontological stipulations

According to [8] we subscribe to the ontological upper-level distinction between *continuants *(also called *endurants*) and *occurrents *(also called *perdurants*). Continuants are characterized as those entities that are wholly present in time, i.e. all their proper parts are present at any time of their existence. Typical continuants are physical objects and spaces, e.g. organisms and anatomical structures. In contrast, occurrents are entities that "happen in time": They extend in time by accumulating different temporal parts, so that, at any time instant *t *at which they exist, only their temporal parts at *t *are present. Typical occurrents are processual entities and events, such as surgical or diagnostic procedures. Whereas there is no doubt that medical prodecures are always occurrents (they have a well-defined beginning and end as temporal parts), diseases and other body phenomena are often ontologically ambiguous. On the one hand, they can be considered as states und thus being categorized as (immaterial) continuants. On the other hand, the focus can be laid on their temporal course, what characterizes them as occurrents. In this paper, this distinction will be neglected.

In the following we will analyze and discuss the formalization of complex SNOMED CT definitions.

## Case studies of complex definitions

Using description logic constructors, SNOMED CT is able to define complex classes which are composed of simpler ones. To this end, the conjoints are grouped in terms of so-called *relationship groups *[9].

Relationship groups had been introduced in order to group attribute-value pairs which are "logically associated with each other" [10]. For example, a "removal of a foreign body from the stomach by gastrotomy" procedure involves the "removal" of a "foreign body" (not of a stomach) and the "incision" of a "stomach" (not of a foreign body). In description logic notation, SNOMED CT uses the symbol rg for relationship groups. It is treated like an existentially quantified role and used for nesting the associated expressions as in:

(1) ExtrOfForeignBodyFromStomachByExcision≡∃rg.(∃ProcedureSite.StomachStructure⊓∃Method.IncisionAction)⊓∃rg.(∃ProcedureSite.StomachStructure⊓∃DirectMorphology.ForeignBody⊓∃Method.RemovalAction)

Thus misinterpretations such as "removal of stomach" and "incision of foreign body" can be prevented. As we have recently analyzed in [11], complex medical procedures or findings are characterized by a mereological structure, i.e. they can be described in terms of their (temporal) parts. As a consequence, rg can be considered equivalent to the partitive relation has-part, which would improve the semantic clarity in these cases. The expression ∃rg.(*A *⊓ *B*) ⊓ ∃rg.(*C *⊓ *D*) is then equivalent to ∃has-part.(*A *⊓ *B*) ⊓∃has-part.(*C *⊓ *D*).

We emphasize that this is a central issue, since complex definitions are frequent in SNOMED CT. Among all active SNOMED CT concepts, approximately 25,000 textual descriptions include one of the words "and", "with", "without". In about one third of these cases, relationship groups are used in the definitions associated.

### Complex definitions with implicit time sequences

Using has-part instead of rg, the representation of the SNOMED CT concept "Extraction of Foreign Body from Stomach by Excision" can be expressed as the following description logic definition:

(2) ExtrOfForeignBodyFromStomachByExcision≡∃has-part.(∃ProcedureSite.StomachStructure⊓∃Method.IncisionAction)⊓∃has-part.(∃ProcedureSite.StomachStructure⊓∃DirectMorphology.ForeignBody⊓∃Method.RemovalAction)

According to [11], rg was substituted by has-part for reasons of clarification. Obviously, *Extraction of Foreign Body from Stomach by Excision *can be fairly well represented using the constructors as introduced for SNOMED CT. However, an in-depth ontological analysis reveals that there are hidden assumptions regarding time that are not adequately expressed.

Let us try to express the same kind of state of affairs using first order logics with equalities and inequalities. We here abstract from the above example, using the constants *C *for a complex occurrent (e.g. *Extraction of Foreign Body from Stomach by Excision*) and *A*_1_, *A*_2 _for atomic occurrents (e.g. *Incision of Stomach, Removal of Foreign Body from Stomach*). Furthermore, we introduce the instantiation relation inst which relates individuals with classes in a comparable way to the standard translation approach given by the model theoretic semantics of description logics [4]. With the standard translation from a description logic class to a first-order formula, we would not need the inst relation because classes are translated to first-order unary predicates (rather than constants) and individuals correspond to constants populating the corresponding classes. However, we adopt this notation for the purpose of consistency with that in [11]. The relation between an occurrent (medical procedure) and its participating continuants (physical objects or anatomical structures) at a point in time *t *is given by the time-indexed relation has-participant (which denotes the time in which each occurrent begins), according to the specification of foundational relations in bio-medical ontologies introduced by [12]. Finally, *S *stands for the class of anatomical structure which participates in the two subprocesses and Δ*t*_max _for the maximal time which separates the begin of the second from that of the first one.

(3) ∀c:inst(c,C)↔∃s,t1,t2,a1,a2:inst(a1,A1)∧inst(a2,A2)∧inst(s,S)∧has-part(c,a1)∧has-part(c,a2)∧has-participant(a1,s,t1)∧has-participant(a2,s,t2)∧|t2−t1|≤Δtmax⁡

This representation is semantically richer insofar as it states that all pairs of subprocesses have the *same *anatomical structure *s *as a participant and that they occur within a pre-defined time interval, e.g. during a hospital stay. In contrast, the description logic representation for this and similar clinical situations would hold true even if there were two different stomachs and also if the two subprocesses were part of two completely different surgical interventions. However, the pragmatics of clinical coding is supposed to prevent such misinterpretations, since the assignment of a clinical code is used to be attributed to a single surgical procedure or a single disease under treatment.

### Complex definitions with explicit time sequences

Let us now analyze a typical complex disease definition, in which a temporal order of the subprocesses is clearly stated such as the SNOMED CT concept "Concussion of brain with loss of consciousness". (Here, interestingly, the original SNOMED CT definition is underspecified, using a primitive class definition (⊑) instead of a full one (≡). Undoubtedly, the given expression fulfills also the sufficient conditions for the class in question.), expressed in description logic as follows:

(4) $$\begin{array}{l} \text{BrainConcussionWithLossOfConsciousness}\equiv \\ \exists \text{has-lart}.\\ (\text{Concussion}⊓\\ \exists \text{FindingSite}\text{.BrainTissueStructure})⊓\\ \exists \text{has-part}\text{.LossOfConsciouness} \end{array}$$

In opposition to the above example, here the sequence of the related events is of utmost importance for correct interpretation. A head injury may be followed by a loss of consciousness, e.g. due to cerebral concussion. The inverse scenario is also possible: a patient loses consciousness (e.g. due to excessive alcohol consumption) and then falls and suffers a head injury. How can we encode this temporal sequence using description logics? Let us propose a modified version of Formula 4 with the help of an irreflexive and transitive relation precedes to relate two occurrents.

(5) $$\begin{array}{l} {\text{BrainConcussionWithLossOfConsciousness}}^{*}\equiv \\ \text{Concussion}⊓\\ \exists \text{FindingSite}\text{.BrainTissueStructure}⊓\\ \exists \text{precedes}\text{.LossOfConsciouness} \end{array}$$

Here we have defined the class of those concussion occurrents characterized by being followed by "loss of consciousness" occurrents (instead of defining a complex occurrent such as in Formula 4). Analogously, one could replace the relation precedes with its inverse follows in order to characterize those cases in which the loss of consciousness has occurred prior to the brain concussion. In principle, there is no reason to give preference to either of these two definitions since the meaning of an expression "*A *with *B*" is ambiguous nevertheless. It can equally be understood as "a kind of *A *which is characterized by being followed by some *B*" or "a composed occurrent consisting of an *A *and a *B*". In principle, combinations are feasible, e.g.

(6) $$\begin{array}{l} {\text{BrainConcussionWithLossOfConsciousness}}^{**}\equiv \\ \exists \text{has-part}.\\ (\text{Concussion}⊓\\ \exists \text{FindingSite}\text{.BrainTissueStructure}⊓\\ \exists \text{precedes}\text{.LossOfConsciouness)}⊓\\ \exists \text{has-part}\text{.LossOfConsciouness} \end{array}$$

Such a definition is, however, affected by the same shortcomings as already discussed with the first example. Firstly, the time interval is not at all specified, and secondly, the definition is also compatible with two separate LossOfConsciousness subprocesses. For the latter problem there is no solution in the description logics used. The first one could pragmatically be tackled by subdividing the temporal relations precedes and follows into two relations which relate to different time intervals. For instance, precedes_curr_/follows_curr _might be introduced do denote the time frame of the current treatment episode, whereas precedes_hist_/follows_hist _would then refer to processes in the context of clinical history only.

Finally, "Brain concussion with loss of consciousness" may only represent a snapshot-like current state of a patient in which no statement about the underlying time-dependent processes are made. This is, however, not sufficient for a final diagnosis, for which a more complete characterization of the disorder or trauma should be expected. The description logic representation used in SNOMED CT does not allow for this distinction. Again, first order logics would be required to describe this in the same way as illustrated in Formula 3, introducing time variables.

### Complex definitions with exclusions

Our third and last example is a modification of the one illustrated by Formula 4. "Concussion of Brain WITH NO Loss of Consciousness" is characterized by the fact that a certain condition (here loss of consciousness) does NOT occur. Aware of the fact that a negation operator is not part of the language specification underlying SNOMED CT we formalize this example based on an existing SNOMED CT definition as follows:

(7) $$\begin{array}{l} \text{BrainConcussionWithNoLossOfConsciousness}\equiv \\ \exists \text{has-part}.\\ (\text{Concussion}⊓\\ \exists \text{FindingSite}\text{.BrainTissueStructure)}⊓\\ \exists \text{has-part}\text{.Awake} \end{array}$$

This representation, again, would be sufficient for recording a snapshot-like state of a patient who is – at the moment of examination – awake and has a clinical picture which is compatible with a brain concussion. However, what is more probably meant here – at least if we use this class in order to express a diagnostic statement – is that the patient has suffered a brain concussion at a certain instant in time (*t*_1_) and that during the whole time interval until the moment of clinical examination (*t*_2_) there was no occurrence of loss of consciousness. The problem with the reference to medical conditions such as Awake and LossOfConsciouness is that both may hold true for parts of this time interval. The patient could have stayed awake immediately after the trauma and then have gradually lost consciousness. Abstracting away from the example, we introduce a complex occurrent *C *each instance of which has an atomic subprocess which is an instance of *A*_1_, but no instance of the subprocess *A*_2_. More precisely: Within a given time interval a patient (an instance of *P*) suffers from a condition of the type *A*_1 _(which has some body structure of type *S*_1 _as participant) without, however, suffering from a condition of the type *A*_2 _located at any *S*_2 _at any time. In the first order logic this can be expressed as follows:

(8) $$\begin{array}{l} \forall c:\text{inst}(c,C)↔\\ \exists a_{1},s_{1},s_{2},p,t_{1}:\text{inst}(a_{1},A_{1})\wedge \text{inst}(p,P)\wedge \\ \text{inst}(s_{1},S_{1})\wedge \text{inst}(s_{2}{\text{,S}}_{2}\text{)}\wedge \text{has-part}(c,a_{1})\wedge \\ \text{has-part}(p,s_{1})\wedge \text{has-part}(p,s_{2})\wedge \\ \text{has-participant}(a_{1},s_{1},t_{1})\wedge \\ \neg (\exists t,a_{2}:t>=t_{1}\wedge \text{inst}(a_{2},A_{2})\wedge \\ \text{has-part}(\text{c},{\text{a}}_{2})\wedge \text{has-participant}(a_{2},s_{2},t)) \end{array}$$

This complex situation is definitely beyond the expressivity of the SNOMED CT description logic, and as a consequence, "Concussion of brain with no loss of consciousness" cannot be pre-coordinated (defined) in SNOMED CT as a fully defined class though the sufficient conditions are completely known. A possible way out could be to enhance the description logic with negation (the negation operator is denoted by ¬) and put it down in the following way:

(9) $$\begin{array}{l} \text{BrainConcussionWithNoLossOfConsciousness}\equiv \\ \exists \text{has-part}.\\ (\text{Concussion}⊓\\ \exists \text{FindingSite}\text{.BrainTissueStructure)}⊓\\ \neg \exists \text{has-part}\text{.LossOfConsciouness} \end{array}$$

Again, here is nothing stated about the referents of "Brain tissue structure" and "Loss of Consciousness". However, the practical usage of clinical terminologies rules out the interpretation that one occurrent has body parts of different patients as participants.

The introduction of the full negation into the set of allowed constructors for SNOMED CT would seriously jeopardize the usability and scalability of terminological reasoning [13].

Nevertheless, it would be crucial not only for correctly defining numerous complex procedures and diseases but also for correctly mapping clinical classification systems such as ICD-10. Here, many classes have clearly defined exclusions (e.g. the general class "Thrombosis" excludes "Thrombosis in Pregnancy") and logical complements (e.g. the ubiquitous "not elsewhere classified" classes). Such categories cannot be adequately represented without full negation [14].

Reification could be a partial solution to the negation problem: with "*Period with no loss of consciousness*" – replacing the class "*Awake*" from Formula 7 – a class is introduced which paraphrases a negative statement without resorting to explicit negation. This class represents a period in the life of a patient during which a loss of consciousness can be ruled out. Again, there is a hidden assumption, *viz*. that a "Period with no loss of consciousness" exactly starts with the traumatic event and ends with the moment in which the observation was made.

SNOMED CT addresses the negation problem by its context model. Although incomplete in some logical respects, it enables support for negation in the sense of "absence of a condition" and "procedure not done", etc. However, we must be aware that this is placed outside the description logics framework. Ignoring this, we would get erroneous conclusions, such as interpreting "ECG not done – Associated Procedure – Electrocardiographic Procedure" as "∃AssociatedProcedure.ElectrocardiographicProcedure". In this case, the quantifier ∃ would assert the existence of some Electrocardiographic Procedure, which is exactly the contrary of what we want to express. Hence, the interpretation of SNOMED CT context model concepts as description logics axioms is not recommended.

## Conclusion

The description of complex medical occurrents such as diseases and procedures is complicated and ambiguous. One source of ambiguity is related to conceptual scope: The same situations can be described as

• a complex occurrent *C *that has *A *and *B *as temporal parts

• a simple occurrent *A' *defined as a kind of *A *followed be some *B*

• a simple occurrent *B' *defined as a kind of *B *preceded be some *A*

In such cases, semantic interoperability would be only achieved in cases that all those mutually dependent classes were instantiated.

Another intricate problem is the reference to occurrents of the type *A *which are characterized by the absence of some occurrent of type *B*. This would require negation, which is not included in the SNOMED CT syntax. A possible way out is the reification of negative statements in the sense of "period with no occurrence of any *B*". Nevertheless, the temporal scope of such complex expressions remains fuzzy. This may be less problematic in practice where clinical codes are generally attributed to well-defined time intervals of treatment or hospitalization episodes. The SNOMED CT context model already addresses this kind of problems.

A more principled way of defining complex occurrents will help improve the quality of the terminology itself in terms of reducing errors and assuring its internal consistency. An assessment of the impact of such improvements on the quality of terminological classification is still speculative for two reasons. Firstly, large scale experiences of the use of description logic-based classification and reasoning are still missing. Secondly, most SNOMED CT descriptions of complex occurrents are still primitive ones, so that misclassifications are not to be supposed.

However, we expect that the issues addressed in this paper will gain relevance along with the maturing of the terminology and the development of more knowledge-intensive applications.

## Competing interests

The authors declare that they have no competing interests.

## Authors' contributions

The first author provided the examples and wrote the first draft of the paper. The other two authors critically assessed the formalizations and contributed to the final redaction of the paper.
